# Protoplasts Isolation and Transient Transformation System Optimization for Poplar 84K (*Populus alba* × *Populus glandulosa*)

**DOI:** 10.3390/biology15100780

**Published:** 2026-05-14

**Authors:** Chao Yu, Huimin Yu, Yirong Rui, Meiling Wang

**Affiliations:** 1 Collaborative Innovation Center for Efficient and Green Production of Agriculture in Mountainous Areas, College of Horticulture, Zhejiang A&F University, Hangzhou 311300, China; 19857196207@163.com (H.Y.); 15268002091@163.com (Y.R.); 2 Key Laboratory of Vegetable Germplasm Innovation and Quality Breeding in the Province, Zhejiang Academy of Agricultural Sciences, Hangzhou 310021, China; 3 College of Agriculture and Biology, Liaocheng University, Liaocheng 252000, China; wangmeiling@lcu.edu.cn

**Keywords:** poplar 84K, protoplast

## Abstract

In poplar, the protracted stable genetic transformation procedure constrains rapid gene functional analyses. To address this limitation, we optimized a protocol for the high-yield isolation and efficient transient transformation of protoplasts from leaves of tissue-cultured poplar 84K (*Populus alba* × *Populus glandulosa*). Through systematic refinement, we determined that an enzymatic digestion solution containing 3% cellulase R-10, 0.3% macerozyme R-10, 0.8% pectolyase R-10, and 0.4 M mannitol was optimal. This formulation, applied over a 3 h digestion period, yielded 12.9 × 10^6^ protoplasts per gram fresh weight, with 93.45% viability. Furthermore, we optimized the parameters for polyethylene glycol -mediated transformation. Using 60 µg of plasmid DNA, 40% polyethylene glycol 4000, and a 20 min incubation, we achieved a high transfection efficiency of 68.67%. The established transient expression system thus provides a reliable, rapid, and effective platform for functional characterization-related studies, such as subcellular localization, protein–protein interactions, and gene expression analyses in poplar, thereby supporting molecular breeding applications.

## Simple Summary

Protoplasts serve as a versatile material for rapid genetic analyses, yet their application in woody species, particularly poplar, remains limited due to genotype-dependent protocols and a low transformation efficacy. In this study, we developed a protocol specifically for poplar 84K (*Populus alba* × *Populus glandulosa*). Through systematic optimization, we identified the best enzymatic formulation and conditions for the rapid, within 3 h, high-yield isolation of viable leaf protoplasts. We further optimized a highly efficient transformation method that introduced a plasmid expressing green fluorescent protein into these cells. This newly established, rapid protoplast system provides a robust platform for expediting the functional characterization of poplar genes, including subcellular localization, protein–protein interactions, and gene expression analyses, directly in poplar cells, thereby significantly advancing molecular breeding and genetic studies in this economically important tree species.

## 1. Introduction

Protoplasts are defined as the intact, living components of a plant cell and are fundamentally characterized by the absence of a cell wall [1]. They harbor the complete genome and, under suitable culture conditions, can regenerate a cell wall and develop into whole plants via either somatic embryogenesis or organogenesis [2]. The removal of the rigid cell wall markedly increases cellular permeability. This enhanced permeability facilitates the efficient introduction of exogenous macromolecules, enabling techniques such as somatic hybridization via protoplast fusion [3] and supporting various genetic manipulations. Collectively, these attributes establish protoplasts as a versatile and powerful material for genetic transformation and functional genomics [4]. Compared to conventional stable transformations, which can take months to generate transgenic lines, protoplast-based systems offer significant experimental advantages. They enable rapid, often high-throughput, functional validations of genes within a matter of hours or a single day [5]. Furthermore, the uniform, single-cell nature of protoplast populations eliminates tissue heterogeneity, allowing for high-resolution analyses of protein subcellular localization [6,7,8] and early signaling events [9,10]. However, it is critically important to acknowledge that protoplasts are isolated single cells that lack a cell wall and intercellular communication. Consequently, findings from this system, particularly those related to protein localization and signaling pathways, require cautious interpretation and must be validated through complementary whole-plant or organ-level studies to avoid potential artifacts [11]. As a result, protoplasts are valuable tools for rapid, initial screenings in applications such as protein interaction analysis [10,12], genome editing [13], and crop trait improvement [14].

However, the broader application of protoplast technology is hampered by substantial variability in isolation and transformation efficiencies across different plant species and genotypes [7]. Although the *Arabidopsis thaliana* mesophyll protoplast system is well-established and widely utilized [15,16], its effectiveness as a heterologous expression platform for genes from other species is limited by the lack of relevant biological context [17]. These constraints stem from several inherent challenges of heterologous protoplast expression that must be carefully considered. These challenges can be broadly categorized into cellular stress responses induced by the isolation and transformation process [18,19], limitations in transfection efficiency and transient expression duration [20], and issues related to proper protein folding, modification, or trafficking [21]. Therefore, establishing a robust, homologous protoplast expression system within a target species is crucial to ensure experimental accuracy and biological relevance.

Protoplast isolation and transient transformation have been reported in several poplar species and hybrids. Guo et al. [11] established a highly efficient leaf mesophyll protoplast protocol for *Populus tremula* × *alba* clone 717-1B4. Yang et al. [22] optimized protoplast preparation from *Populus simonii* × *Populus nigra* leaves, achieving a yield of 2.1 × 10^7^ protoplasts/g fresh weight (FW), with 98.3% viability. Yang et al. [23] developed a system for *Populus* × *xiaohei* using 2% cellulase R-10 and 0.4% macerozyme R-10. Most recently, Tang et al. [24] compared isolation systems across seven poplar genotypes from four taxonomic sections, demonstrating that enzyme composition must be tailored to genotype. Despite these advances, key issues remain unresolved. Protoplast yield, viability, and transformation efficiency vary significantly among genotypes due to differences in cell wall composition and leaf ontogeny [3,7]. A universally applicable protocol is still lacking, and the ionic compositions of post-transfection buffers (e.g., W5 vs. WI) have not been systematically evaluated for long-term cultures, potentially affecting the reliability of downstream assays.

Protoplast isolation methods are broadly categorized into mechanical and enzymatic approaches. Compared to mechanical methods, enzymatic digestion provides milder conditions, yielding protoplasts with superior membrane integrity, higher viability, and greater yield, making it the predominant technique [25]. Protoplast research in woody species has been relatively slow [3,26]. This slower progress is primarily attributed to the intrinsic biological constraints of woody species, which include long life cycles, complex secondary metabolism, and often recalcitrant responses to in vitro culturing and regeneration [26].

Poplar is an economically important tree species worldwide. Its rapid growth and high biomass yield make it a key resource for timber, as well as pulp and paper, and a promising feedstock for lignocellulosic biofuel [11,22,23,24]. The 84K poplar (*Populus alba* × *Populus glandulosa*) has emerged as a prominent model system for functional genomics in woody plants, owing to its rapid growth, high-quality reference genome, and well-established *Agrobacterium*-mediated genetic transformation protocols [27]. This hybrid clone is widely cultivated across northern China and is used to investigate fundamental biological processes such as developmental regulation, abiotic and biotic stress responses, and intracellular signal transduction pathways [28,29]. However, stable genetic transformation in 84K poplar and other *Populus* genotypes still faces challenges, including lengthy timelines and variable efficiencies [27,30,31]. These limitations constrain rapid gene function characterization and impede comprehensive functional genomics studies [23]. Therefore, protoplast-based transient expression systems offer a rapid, genotype-flexible, and high-throughput complementary approach that can greatly expedite the initial stages of gene functional characterization, particularly for subcellular localization, protein–protein interaction, and gene expression analyses [22,23]. Thus, protoplast-based transient expression systems have gained recognition as forming a powerful complementary approach [8]. The efficacies of protoplast isolation and transient expression are highly genotype-specific, necessitating systematic optimization for each species or cultivar [8,32]. This variability arises from genotypic differences in cell wall composition, endogenous enzyme activities, osmotic sensitivity, and cellular repair mechanisms.

In this study, we aimed to address these problems by developing and optimizing protoplast isolation and transient transformation protocols for 84K poplar, and by systematically evaluating key parameters affecting yield, viability, and transformation efficiency.

## 2. Materials and Methods

### 2.1. Plant Material Preparation

Stem segments of 84K poplar (*P. alba* × *P. glandulosa*) were surface-sterilized and cultured on shoot proliferation medium containing Murashige and Skoog medium [33] supplemented with 0.1 mg·L^−1^ α-naphthaleneacetic acid and 0.02 mg·L^−1^ thidiazuron. After 30 days of culturing, axenic plantlets were obtained. These plantlets were then transferred to rooting medium, consisting of half-strength Murashige and Skoog medium with 0.05 mg·L^−1^ α-naphthaleneacetic acid, and grown under a 16 h light/8 h dark photoperiod at 500–600 μmol m^−2^ s^−1^ photosynthetic photon flux density for 30 days.

### 2.2. Chemicals and Solution Preparation

Key chemicals used in this study included cellulase R-10 and macerozyme R-10, pectolyase R-10 (Kyowa Hakko Kirin Co., Ltd., Tokyo, Japan), polyethylene glycol (PEG) 4000, MES, and D-glucose (Sigma-Aldrich, St. Louis, MO, USA), BSA (Amresco, Solon, OH, USA), as well as D-mannitol, KCl, NaCl, MgCl_2_·6H_2_O, and CaCl_2_ (Biodee Biotechnology Co., Ltd., Beijing, China).

### 2.3. Enzyme Digestion Solution

The digestion solution contained 20 mM KCl, 20 mM MES (pH 5.7), 10 mM CaCl_2_, and 0.10% BSA as constant components. The concentrations of D-mannitol, cellulase R-10, macerozyme R-10, and pectolyase Y-10 were optimized through single-factor experiments followed by An L_9_(3^4^) orthogonal array tests (see Table 1 for specific factor levels).

### 2.4. Optimization of Protoplast Isolation Conditions

To identify the optimal concentrations of cellulase, macerozyme, pectolyase, and mannitol, single-factor experiments were initially performed. A baseline enzyme digestion solution, containing 4% (*w*/*v*) cellulase R-10, 0.4% (*w*/*v*) macerozyme R-10, 0.8% (*w*/*v*) pectolyase R-10, 0.4 M mannitol, 20 mM KCl, 20 mM MES (pH 5.7), 10 mM CaCl_2_, and 0.1% (*w*/*v*) BSA, was used for a 4 h digestion at 23 °C. The concentration of only one component was varied at a time within the following ranges: cellulase, 1–6%; macerozyme, 0.1–0.6%; pectolyase, 0.4–0.9%; or mannitol, 0.1–0.6 M, while keeping the other components constant. Based on the single-factor experiment results, three favorable levels for each factor were selected. An L_9_(3^4^) orthogonal array design was then applied to systematically optimize these four factors, resulting in nine distinct treatment combinations (see Table 1). To determine the optimal digestion time, treatments of 1, 2, 3, 4, and 5 h were tested using the optimized enzyme solution.

### 2.5. Protoplast Isolation

Protoplasts were isolated from young leaves of in vitro cultured 84K poplar seedlings using a modified enzymatic digestion protocol [11,34]. Leaf tissue was cut into thin strips (~1 mm wide) and digested in the optimized enzyme solution in the dark at 23 °C for 5–7 h with gentle shaking (10 rpm). The digested mixture was passed through a 400-mesh cell strainer to remove debris. Protoplasts were pelleted by centrifugation (100× *g*, 5 min, 23 °C) and washed twice with 2 mL of ice-cold W5 solution [154 mM NaCl, 12.5 mM CaCl_2_, 5 mM KCl, and 2 mM MES (pH 5.7)] at 100× *g* and 4 °C for 5 min. The pellet was then resuspended in 10 mL of ice-cold W5 solution and kept on ice for 30 min. After a final centrifugation under the same conditions, protoplasts were resuspended in ice-cold MMG solution [0.4 M D-mannitol, 15 mM MgCl_2_, and 4 mM MES (pH 5.7)]. Protoplast concentration and viability were determined using an XB-K-25 hemocytometer and fluorescein diacetate staining, respectively. The latter was performed using a 0.1% (*w*/*v*) working concentration incubated for 5 min at room temperature in the dark. All the experiments were performed with at least three biological replicates.

### 2.6. PEG-Mediated Protoplast Transformation

A PEG-mediated transient transformation assay was performed to introduce a plasmid carrying the green fluorescent protein (GFP) reporter gene under the control of the CaMV 35S promoter, pBinGFP, into poplar protoplasts to systematically evaluate and optimize the transformation efficiency. In total, 10 µL of plasmid DNA (20, 40, 60, or 80 µg) was gently mixed with 100 µL of protoplast suspension (1 × 10^5^ protoplasts/mL) in a 1.5 mL tube. An equal volume (110 µL) of PEG/Ca^2+^ solution [pH 5.7, containing 30%, 40%, 50%, or 60% PEG 4000 (*w*/*v*) PEG 4000, 0.2 M D-mannitol, and 0.1 M CaCl_2_] was added, and the mixture was gently stirred using a wide-bore tip. The mixture was then incubated in the dark at 23 °C for varying durations (10, 15, 20, or 30 min). The reaction was stopped with 1 mL of W5 solution, followed by centrifugation at 100× *g* for 5 min at 23 °C. After discarding the supernatant, the washing step was repeated once. The protoplast pellet was resuspended in 1 mL of W5 solution and incubated in the dark at 23 °C for 12–16 h. Finally, the sample was centrifuged at 100× *g* for 1 min at 23 °C, the supernatant was removed, and the protoplasts were resuspended. An aliquot was mounted on a slide and observed under a confocal laser scanning microscope (FV3000, Olympus, Tokyo, Japan). For GFP, the excitation wavelength was set to 488 nm, with emission collected over a range of 505–545 nm. For Cy5 fluorescence, the excitation wavelength was set to 640 nm, with emission collected over a range of 650–750 nm. Images were processed using Adobe Photoshop software (version 21.0.2). Each experimental condition was performed with three independent biological replicates.

### 2.7. Statistical Analysis

The statistical analysis was performed using SPSS Statistics software (version 27.0.1). The statistical significance of differences among treatment means for measured parameters was tested using a one-way analysis of variance. GraphPad Prism software (version 10.1.2) was used for generating graphs. All the experiments included at least three independent biological replicates, and data are presented as means ± standard deviations.

## 3. Results

### 3.1. Optimization of Plant Material for Protoplast Isolation

**Figure 1:**
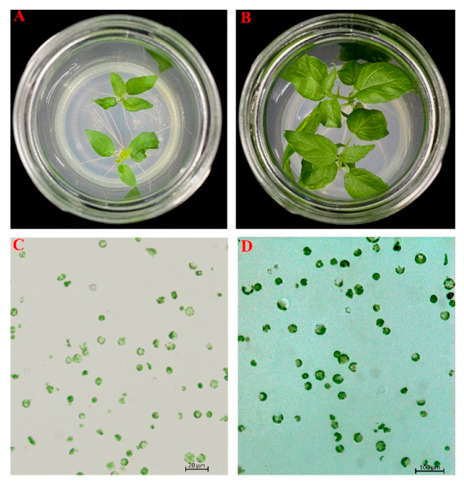
Effect of leaf age on the efficiency of protoplast isolation from 84K poplar leaves. (**A**) 7-day-old tissue-cultured seedlings. (**B**) 30-day-old tissue-cultured seedlings. (**C**) Visualization of protoplasts of 7-day-old tissue-cultured seedlings. (**D**) Visualization of protoplasts of 30-day-old tissue-cultured seedlings.

The age and developmental stage of the source tissue significantly influenced protoplast yield and quality. Leaves harvested from 7-day-old tissue-cultured seedlings were small, tender, and light green, yielding protoplasts that were generally small and often fragmented (Figure 1A,C). In contrast, leaves from 30-day-old seedlings were dark green and fully expanded, consistently producing spherical, intact protoplasts with the highest yield (Figure 1B,D). Therefore, leaves from 30-day-old tissue-cultured seedlings were identified as the optimal material for high-yield and high-quality protoplast isolation in subsequent experiments.

### 3.2. Optimization of Enzymatic and Osmotic Conditions via Single-Factor Experiments

**Figure 2:**
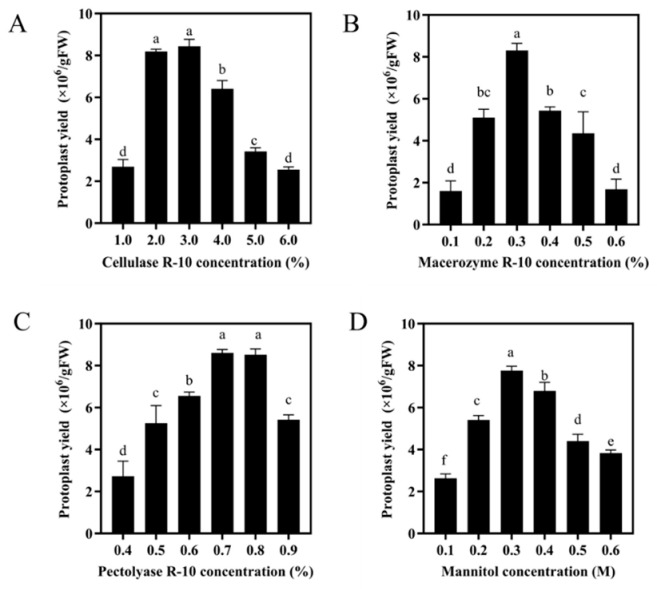
Effects of single factors on the yield of 84K poplar protoplasts. (**A**) Cellulase R-10 (%). (**B**) macerozyme R-10 (%). (**C**) pectolyase R-10 (%). (**D**) mannitol (M) concentrations on the yield of protoplasts from leaves. Different lowercase letters indicate significant differences between samples by Duncan’s multiple range tests at *p* < 0.05. Bars represent standard errors (SE).

We first evaluated the effects of cellulase R-10, macerozyme R-10, pectolyase R-10, and mannitol concentrations on the yield of protoplasts isolated from poplar leaves. As shown in Figure 2, for each factor, protoplast yield initially increased along with concentration, reached a maximum, and then declined at higher levels. Insufficient concentrations of enzymes or osmotica resulted in incomplete protoplast release, whereas excessively high concentrations led to significant yield reductions. These results indicated the existence of an optimal concentration for each component to maximize protoplast yield.

**Figure 3:**
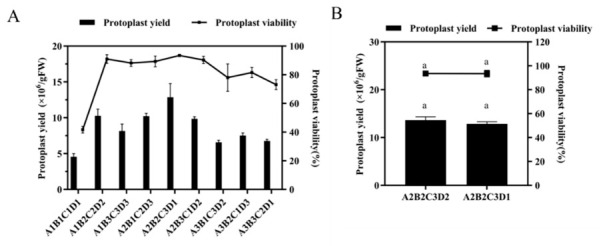
Effects of different treatment conditions on protoplast yield and viability from 84K poplar. Changes and Statistical Analysis. (**A**) Effects of L_9_(3^4^) orthogonal array on protoplast yield and viability. (**B**) Verification test of the optimal conditions for protoplast yield and viability. The same lowercase letter above the bars indicates no significant difference among treatments at *p* < 0.05 level, as determined by Duncan’s multiple range test.

Based on the peaks identified in the single-factor experiments, three levels for each factor were selected for subsequent orthogonal array optimization: cellulase R-10 at 2%, 3%, and 4% (*w*/*v*); macerozyme R-10 at 0.2%, 0.3%, and 0.4% (*w*/*v*); pectolyase R-10 at 0.6%, 0.7%, and 0.8% (*w*/*v*); and mannitol at 0.3 M, 0.4 M, and 0.5 M (Figure 2). To investigate the effects of four key factors, cellulase R-10, macerozyme R-10, pectolyase R-10, and mannitol concentration, on protoplast yield and viability, an L_9_(3^4^) orthogonal array design was employed (Table 1). The experimental results are summarized in Supplementary Table S1. A range analysis indicated that the order of factors influencing protoplast yield was cellulase R-10 > macerozyme R-10 > mannitol > pectolyase R-10, whereas for viability the order was cellulase R-10 > macerozyme R-10 > pectolyase R-10 > mannitol. The orthogonal experiment results depict the trends of each response variable across the tested factor levels (Figure 3A). Based on this analysis, the predicted optimal combination was (cellulase R-10)_2_ (macerozyme R-10)_2_ (pectolyase R-10)_3_ (mannitol)_2_, corresponding to 3% (*w*/*v*) cellulase R-10, 0.3% (*w*/*v*) macerozyme R-10, 0.8% (*w*/*v*) pectolyase R-10, and 0.4 M mannitol, a combination not originally included in the orthogonal design. Notably, within the orthogonal design, the highest yield and viability among all the tested combinations were achieved with 3% cellulase R-10, 0.3% macerozyme R-10, 0.8% pectolyase R-10, and 0.3 M mannitol. Consequently, a verification experiment was conducted to compare this best-performing combination from the orthogonal test with the statistically predicted optimum. As shown in Figure 3B, the predicted combination (with 0.4 M mannitol) yielded 12.9 × 10^6^ protoplasts/g FW, with 93.45% viability. Although this yield was marginally higher than that obtained with the 0.3 M mannitol combination, the difference was not statistically significant (Figure 3B). Therefore, the final optimized enzymatic digestion solution was established as 3% (*w*/*v*) cellulase R-10, 0.3% (*w*/*v*) macerozyme R-10, 0.8% (*w*/*v*) pectolyase R-10, and 0.4 M mannitol.

**Figure 4:**
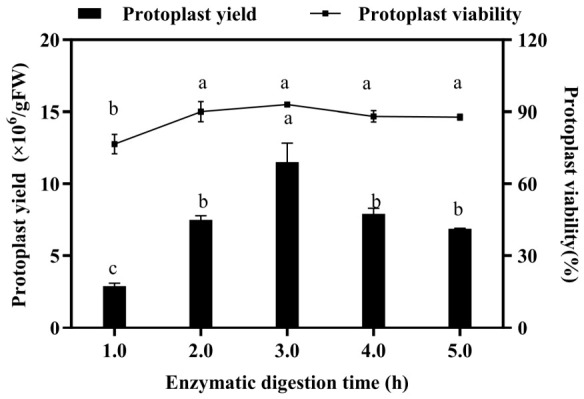
The effect of enzymatic digestion time (h) on the yield and viability of 84K poplar protoplasts. Different lowercase letters indicate significant differences between samples by Duncan’s multiple range tests at *p* < 0.05. Bars represent standard errors (SE).

Using this optimized enzymatic formulation, digestion times ranging from 1 to 5 h were evaluated. Protoplast yield increased with digestion time, plateauing after 3 h without further significant improvement; conversely, extending digestion to 5 h resulted in a decline in yield (Figure 4). In contrast, protoplast viability remained consistently high across all the time points, with no significant variation. Based on these findings, a 3 h digestion period was selected as optimal for subsequent experiments.

### 3.3. Optimization of Transient Transformation Conditions for Poplar Protoplasts

Building upon the established efficient protoplast isolation system, we employed a PEG-mediated transformation approach to systematically evaluate the effects of plasmid DNA amount, PEG 4000 concentration, and transformation time on the transient transformation efficiency of poplar protoplasts. Protoplasts were transfected with a plasmid expressing the GFP reporter gene under the control of the CaMV 35S promoter. Initial transformation attempts yielded efficiencies less than 60%, accompanied by high proportions of cell death and fragmentation. To improve these outcomes, the individual effects of each key parameter were investigated.

**Figure 5:**
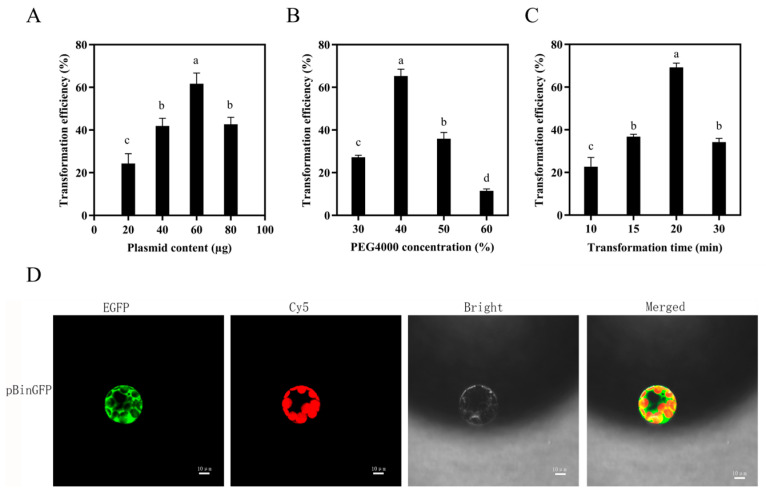
Effects of different factors on the transformation efficiency of 84K poplar. (**A**) Plasmid content (µg). (**B**) PEG4000 concentration. (**C**) Transformation time (min). Different lowercase letters indicate significant differences between samples by Duncan’s multiple range tests at *p* < 0.05. (**D**) GFP expression in 84K poplar protoplasts. The GFP signals is false-colored green and the chloroplast autofluorescence is shown in red. Bars represent standard errors (SE).

First, we tested four different plasmid DNA amounts, 20, 40, 60, and 80 µg, while maintaining the PEG 4000 concentration at 40% and the transformation time at 15 min. The results indicated that transformation efficiency initially increased with plasmid amount, peaking at 60 µg, with approximately 61.67% of protoplasts exhibiting successful GFP expression, before declining at higher amounts (Figure 5A).

To determine the optimal PEG 4000 concentration, we compared transformation efficiencies at 30%, 40%, and 50% (*w*/*v*) PEG 4000. Both 30% and 50% PEG 4000 resulted in significantly lower transformation efficiencies compared to 40% PEG 4000 (Figure 5B), supporting the selection of 40% PEG 4000 as the optimal condition.

Subsequently, with the PEG 4000 concentration fixed at 40%, we evaluated different transformation durations (10, 15, 20, and 30 min). The highest transformation efficiency was achieved with a 20 min incubation, resulting in approximately 69.21% of protoplasts successfully expressing GFP (Figure 5C). Representative confocal laser scanning microscopy images confirmed robust GFP expression in protoplasts following this optimized 20 min transfection with 40% PEG 4000 (Figure 5D).

## 4. Discussion

The 84K poplar (*P. alba* × *P. glandulosa*) is an economically and ecologically important tree species in northern China and serves as a model system for woody plant research [29]. However, its genetic transformation remains inefficient and genotype-dependent, which constrains functional genomics and breeding applications [35]. To address this limitation, we developed and optimized a protocol for the high-yield isolation and transient transformation of protoplasts from leaves of tissue-cultured 84K poplar plantlets. Poplar cell walls are rich in cellulose, hemicellulose, and pectin. The particularly high pectin content contributes to their mechanical strength but also makes enzymatic digestion challenging [36,37]. To effectively degrade these components, we chose a combination of cellulase R-10 (for cellulose degradation), macerozyme R-10 (for pectin and hemicellulose degradation), and pectolyase (for more specific pectin degradation). This enzymatic cocktail has been widely used in protocols for protoplast isolation from recalcitrant woody tissues [11,22].

The fundamental principle of plant protoplast isolation is well-established, but a universal method of preparation is lacking due to significant variations in plant genotypes and tissue specificity [3,23]. Leaf tissues are often the preferred material for protoplast isolation because they contain abundant cells with intact structures and cell walls that are relatively amenable to enzymatic digestion [3,23]. Furthermore, using leaves from sterile, in vitro cultured seedlings eliminates the need for surface sterilization and minimizes microbial contamination. The developmental stage of the leaf is a critical factor: very young leaves contain small cells that are highly sensitive to enzyme toxicity, which can compromise protoplast viability, whereas older leaves exhibit reduced metabolic activity and possess more recalcitrant cell walls. Consequently, neither extreme is ideal for generating high-quality protoplasts [3]. For 84K poplar, we determined that young leaves harvested from 30-day-old tissue-cultured seedlings provided the optimal balance, yielding protoplasts having a uniform physiological state and high viability (Figure 1).

Enzymatic digestion is the prevailing method for plant protoplast preparation, favored for its simplicity, efficiency, and broad applicability [3]. However, due to significant variations in cell wall composition across different plant species and even cultivars, extraction and culture conditions are not universally transferable [38]. For instance, Jenes and Pauk [39] successfully isolated protoplasts from only 20 out of 44 tested rice genotypes, underscoring the genotype-specific nature of optimal digestion conditions.

Following the selection of a suitable source material, the composition and concentration of the enzymatic digestion solution require optimization. Commonly used enzymes include cellulase, macerozyme, and pectolyase. Cellulase degrades cellulose in the primary cell wall, macerozyme promotes tissue maceration and cell separation, and pectolyase breaks down pectin in the middle lamella, thereby enhancing cell dispersal [40]. Optimizing the combination and ratio of these enzymes can significantly improve cell wall digestion efficiency, leading to enhanced protoplast yield and quality. Given the inherent heterogeneity in cell wall structure and composition among different plants and cultivars, it is essential to systematically optimize enzyme types, ratios, and incubation conditions (e.g., temperature, pH, and duration) for specific experimental materials [6,38].

Furthermore, maintaining osmotic balance is critical, because protoplasts are highly susceptible to rupture in the absence of a stable osmotic environment [41]. Osmotic stabilizers, such as D-mannitol, are routinely added to the digestion medium to prevent protoplast swelling and bursting. An insufficient mannitol concentration fails to maintain osmotic equilibrium, leading to membrane rupture and reduced yield, whereas an excessively high concentration induces protoplast plasmolysis and shrinkage, which can impair cellular metabolism and physiological functions [12].

This study employed an orthogonal design with four factors, cellulase R-10, macerozyme R-10, pectolyase R-10, and mannitol concentration, to systematically investigate their effects on protoplast yield (Table 1). The results demonstrated that protoplast yield increased with higher macerozyme concentrations but decreased with higher cellulase concentrations (Figure 2). This dual effect can be attributed to enzyme concentration dynamics: insufficient cellulase fails to adequately degrade the cell wall, limiting protoplast release, while insufficient macerozyme reduces tissue dissociation. Conversely, excessively high concentrations of both enzymes may cause over-digestion of the cell wall, damaging protoplast membrane integrity and ultimately reducing yield [11].

Digestion time is another critical factor influencing isolation efficiency, with the optimal duration varying significantly among plant species and tissues. For instance, the optimal digestion time for fir leaves is 2 h, whereas reports for poplar species are inconsistent. In this study, protoplast yield increased significantly with digestion time, reaching an optimum at 3 h, after which it plateaued (Figure 4).

Common methods for introducing foreign genes into protoplasts include PEG-mediated transformation, electroporation, and microinjection [34]. Among them, PEG-mediated transformation is favored for its simplicity, low cost, and ability to produce a relatively high number of transformed cells. However, its transient transformation efficiency is influenced by multiple parameters, including plasmid DNA concentration, PEG concentration, and transformation duration [13,32].

For plasmid concentration, higher amounts generally promote higher transformation efficiencies. This study systematically tested different plasmid quantities, confirming that efficiency increased along with the amount used, which aligned with previous reports indicating a saturation point beyond which no significant further improvement occurs [3].

PEG functions as both a fusogen and an osmotic stabilizer during protoplast transformation. Inadequate concentrations or low molecular weights result in poor membrane fusion and increased protoplast rupture, whereas excessively high concentrations induce protoplast shrinkage, reduce fusion frequency, and exert cytotoxic effects. Consequently, selecting an optimal PEG concentration is critical for achieving a high transformation efficiency. In this study, the transformation efficiency exhibited a bell-shaped response to the increasing PEG concentration, initially rising and then declining (Figure 5B). At PEG 4000 concentrations of 60% (*w*/*v*) and above, protoplasts displayed obvious deformations or ruptured, which was likely attributable to structural damage caused by the hypertonic stress of high PEG concentrations [42]. Hu et al. [40] reported that a 40% (*w*/*v*) PEG 4000 concentration with a 15 min incubation yielded the highest transformation efficiency in *Eucommia ulmoides* protoplasts, highlighting interspecific differences in the sensitivity to PEG-induced osmotic pressure among woody plants. While PEG6000 has been used in some studies [43,44], we chose PEG4000 in this study as it is the most widely adopted molecular weight for protoplast transformation in model plants such as *Arabidopsis* [34] and rice [45], as well as in poplar [22,23,24]. Although a systematic comparison of different PEG molecular weights was beyond the scope of this work, our results demonstrate that PEG4000 is effective for 84K poplar protoplast transformation.

Selecting an appropriate incubation duration is equally crucial for maximizing transformation efficiency while minimizing PEG-mediated toxicity. By systematically testing different incubation times, this study evaluated their impact on the transient transformation of 84K poplar protoplasts. The results demonstrated that efficiency initially increased and then decreased with prolonged treatment. When incubation exceeded 30 min, microscopic observations revealed abundant cellular debris and a significant reduction in the intact protoplast number. This pattern likely reflected that insufficient time prevents effective plasmid entry, whereas excessive exposure exacerbates PEG-induced cellular damage and lysis, leading to a substantial decline in transformation rate [3,32]. We also note several limitations of the current protocol. W5 solution was designed specifically for protoplast isolation. In this solution, high Cl^−^ maintains cell turgor, while Na^+^ stabilizes vacuolar pH and prevents rapid cell expansion. However, W5 is not an ideal buffer for long term incubation. Its ionic composition is physiologically unbalanced and lacks essential cofactors such as K^+^ and Mg^2+^ required for normal metabolism. Prolonged incubation in W5 for 12 to 16 h can lead to ion starvation, carbon and energy starvation, and failure to restore normal cellular function after the stress of isolation. This is particularly critical for studies involving protein subcellular localization, which depends on intact protein–protein interaction and proper cytoplasmic structure. Neither can be assumed under non-physiological ionic conditions. Therefore, although our system is suitable for rapid, qualitative screening, results from any assay including subcellular localization should be interpreted with caution. Prolonged incubation in W5 may prevent full restoration of cellular function, including the protein–protein interactions and cytoplasmic structure essential for accurate protein localization. For quantitative or long-term studies, future work should adopt a more physiologically balanced buffer containing all 12 essential ions, 0.5 M mannitol as an osmotic stabilizer, and a suitable carbohydrate source. This approach would better support cell function and enable prolonged culture. Post-transformation protoplast viability should be routinely quantified in future studies using well-established methods, such as fluorescein diacetate staining, to support the objective evaluation of protocol stringency and functional assay compatibility. Compared with previous studies on poplar protoplast isolation, our optimized protocol shows clear advantages. For example, Tang et al. [24] reported protoplast yields ranging from 8.59 to 28.10 × 10^6^ protoplasts/g FW across seven poplar genotypes using Enzyme Solution I (1.5% cellulase R-10 + 0.5% macerozyme R-10), with viability varying from 11.28% to 93.87% depending on the genotype. In contrast, our protocol for 84K poplar achieved a yield of 12.9 × 10^6^ protoplasts/g FW, with 93.45% viability, demonstrating the greater stability and reproducibility for this specific genotype. Similarly, Guo et al. [11] obtained approximately 1 × 10^7^ protoplasts/g FW from *P. tremula* × *alba* leaves using 3% cellulase R-10 and 0.8% macerozyme R-10, with a transformation efficiency reaching approximately 50%. Our system achieved a transformation efficiency of up to 68.67%, representing a notable improvement. Yang et al. [22] reported a yield of 2.1 × 10^7^ protoplasts/g FW from *P. simonii* × *P. nigra*, with 98.3% viability, which is comparable to our results, and a transformation efficiency of 17.07%. These comparisons highlight that our protocol provides a standardized, efficient system specifically optimized for 84K poplar, a widely used model genotype that previously lacked a dedicated protoplast protocol. In summary, this study successfully developed an optimized protocol for the high-yield isolation and efficient transient transformation of protoplasts from leaves of tissue-cultured poplar 84K.

## 5. Conclusions

This study developed an optimized protocol for the high-yield isolation and efficient transient transformation of protoplasts from leaves of 84K poplar (*P. alba* × *P. glandulosa*). The optimal conditions were identified as follows: young leaves from 30-day-old tissue-cultured seedlings as source material; an enzymatic solution of 3% cellulase R-10, 0.3% macerozyme R-10, 0.8% pectolyase R-10, and 0.4 M mannitol with 3 h digestion; and a transformation protocol using 60 µg plasmid DNA, 40% PEG 4000, and a 20 min incubation, achieving a 69.21% efficiency. This optimized protocol addresses the key bottleneck of inefficient and genotype-dependent transformation in this model tree species. The reproducible high yield and transformation efficiency form a robust platform to support rapid molecular characterization of genes in woody plants.

## Figures and Tables

**Table 1:** Orthogonal experimental factor level table.

Level	Factors			
	Cellulase R-10 (%)	Macerozyme R-10 (%)	Pectolyase R-10 (%)	D-Mannitol
1	2	0.2	0.6	0.3
2	3	0.3	0.7	0.4
3	4	0.4	0.8	0.5

## Data Availability

The data generated and/or analyzed during this study are available from the corresponding author on reasonable request.

## Supplementary Materials

The following supporting information can be downloaded at: https://www.mdpi.com/article/10.3390/biology15100780/s1, Table S1: The L_9_(3^4^) orthogonal array affecting of protoplast isolation.
