# Supplementary material for: Protoplasts Isolation and Transient Transformation System Optimization for Poplar 84K (Populus alba × Populus glandulosa)
Source: Biology (Basel). 2026 May 14;15(10):780. doi: 10.3390/biology15100780 (PMC13203830; doi:10.3390/biology15100780)
Supplement: Supplementary file 1 [file biology-15-00780-s001.zip › biology-4297353-supplementary.pdf]

Supplementary Materials

Supplementary Table 1 The $L_9(3^4)$ orthogonal array affecting of protoplast isolation

Level	factors				Protoplast yield (× 10^6 /gFW)	Protoplast viability(%)
	A cellulase R-10 (%)	B macerozyme R-10 (%)	C pectolyase R-10 (%)	D mannitol (M)		
A1B1C1D1	2	0.2	0.6	0.3	4.56±0.43	41.67±2.31
A1B2C2D2	2	0.3	0.7	0.4	10.27±0.93	90.94±3.02
A1B3C3D3	2	0.4	0.8	0.5	8.15±0.98	88.20±2.31
A2B1C2D3	3	0.2	0.7	0.5	10.22±0.38	89.30±3.76
A2B2C3D1	3	0.3	0.8	0.3	12.85±1.90	93.45±0.51
A2B3C1D2	3	0.4	0.6	0.4	9.86±0.27	90.33±2.52
A3B1C3D2	4	0.2	0.8	0.4	6.57±0.30	78.00±9.54
A3B2C1D3	4	0.3	0.6	0.5	7.53±0.35	81.50±3.50
A3B3C2D1	4	0.4	0.7	0.3	6.78±0.21	73.12±3.46
Protoplast yield	K1	22.98	21.35	21.95	24.19	
	K2	32.93	30.65	27.27	26.7	
	K3	20.88	24.79	27.57	25.9	
	k1	7.66	7.12	7.32	8.06	
	k2	10.98	10.22	9.09	8.9	
	k3	6.96	8.26	9.19	8.63	
	Range	4.02	3.1	1.87	0.87	
	Rank	A>B>C>D				
	Optimal combination	A2B2C3D2				
Protoplast viability	K1'	220.81	208.97	213.5	208.27	
	K2'	273.08	265.89	253.36	259.27	
	K3'	232.62	251.65	259.65	259	
	k1‘	73.6	69.65	71.17	69.41	
	k2’	91.03	88.63	84.45	86.42	
	k3‘	77.54	83.88	86.55	86.33	
	Range	17.43	18.97	15.38	17.01	
	Rank	B>A>D>C				
	Optimal combination	A2B2C3D2				
